# Supplementary material for: External gas-assisted mold temperature control and optimization molding parameters for improving weld line strength in polyamide plastics
Source: PLoS One. 2024 Aug 22;19(8):e0307485. doi: 10.1371/journal.pone.0307485 (PMC11340943; doi:10.1371/journal.pone.0307485)
Supplement: Attachment — Submitted filename: Cover letter Reviewer 2.docx [file pone.0307485.s001.docx]

**RESPONSE LETTER**

**Ms. Ref. No.:** PONE-D-24-07210

**Journal:** PLOS ONE

**Title:** External gas-assisted mold temperature control and optimization molding parameters for improving weld line strength in polyamide plastics

**Authors:** Nguyen Truong Giang^1,2^, Pham Son Minh^3^, Tran Anh Son^1,2^, Van-Thuc Nguyen^3^, Tran Minh The Uyen^3^, Thanh Trung Do^3^, Van Thanh Tien Nguyen^4*^

Dear Reviewer,

We thank you for your suggestions. The manuscript has been revised according to the reviewer’s comments. The changes made are listed as follows and marked with comment text in the manuscript.

**Reviewer's comments:**

**Reviewer #2:** The authors have explored an injection molding strategy focused on enhancing weld line strength and ductility in PA6 composite samples through elevated mold temperatures. They investigated the impact of different injection molding parameters and conducted further optimization using the ANN-GA approach. However, while the study is intriguing, it lacks clarity, depth, and adequate information in various sections of the manuscript.MAJOR REVISIONS are necessary as detailed below before considering it for publication in the journal.

1. In the abstract, the statement "To optimize the molding parameters, we apply an Artificial Neural Network (ANN) in conjunction with a Genetic Algorithm (GA), marking a sophisticated leap in precision and efficiency over standard optimization methods" requires clarification. Were the results compared with those obtained from other optimization algorithms?

We thank you very much for your suggestion.

This sentence is not suitable, therefore, we have changed it to the better one. We did not compare it to the other optimization algorithms.

We have modified the abstract section as follows:

*+ On page 2 lines 8-10 to read as:*

To optimize the molding parameters, we apply an Artificial Neural Network (ANN) in conjunction with a Genetic Algorithm (GA).

2. “The viscosity of the injection plastic rises, leading to a higher resistance during the molding process.” What resistance? Clarify.

We thank you very much for your suggestion.

We have clarified this sentence, it is the fluid’s resistance to flow.

We have modified the introduction section as follows:

*+ On page 3 lines 3-4 to read as:*

The viscosity of the injection plastic rises, leading to a higher fluid’s resistance to flow during the molding process.

3. The sentence “Besides, increasing the mold temperature by using a mold temperature control system' lacks clarity and completeness. It's unclear what exactly this line signifies. There are multiple sentences of similar nature throughout the manuscript that require clearer expression.

We thank you very much for your suggestion.

This sentence is not clear, we have modified it as follows:

*+ On page 3 lines 7-8 to read as:*

Besides, increasing the mold temperature by using a mold temperature control system is also a solution to improve the filling rate by slowing down the plastic cooling process.

4. "Have the authors prepared Figure 1? If not, it should be properly cited. The figure itself lacks clarity."

We thank you very much for your suggestion.

This figure was prepared by ourselves, and it is further explained in the manuscript.

We have modified the manuscript as follows:

*+ On page 4 lines 4-9 to read as:*

A weld line in injection molding is generated when two molten polymers stream meat in opposite directions, as shown in Fig. 1. Besides the skin layer that reduces the filling rate during the injection molding process, the cooled-down polymer also contributes to the formation of a weld line, as shown in Fig. 1(a). With mold temperature control assistance, the skin layer is thinner, and the plastic temperature at the weld line is higher, which could lead to better bonding, as shown in Fig. 1(b).

5. Was the proposed 'novel Cavity' designed by the authors? Clarify.
"Numerous studies have explored mold temperatures exceeding 100°C. For example, a manuscript published in 2013:
"Meister, S. and Drummer, D.. "Influence of Mold Temperature on Mold Filling Behavior and Part Properties in Micro Injection Molding" International Polymer Processing, vol. 28, no. 5, 2013, pp. 550-557. <https://doi.org/10.3139/217.2804>"

We thank you very much for your question.

We have cited this paper in our manuscript. We referred to this report; we are not the authors of this report.

We have modified the introduction section as follows:

*+ On page 5 lines 16-18 to read as:*

This is facilitated by a newly designed and manufactured mold structure that enables localized heating, termed the 'novel cavity', as introduced by Meister et al. [21].

[21] Meister, S., & Drummer, D. (2013). Influence of mold temperature on mold filling behavior and part properties in micro injection molding. *International Polymer Processing*, *28*(5), 550-557.

6. "The novelty of the work remains unclear and should be distinctly highlighted in relation to the presented literature review. Additionally, many sentences in the introduction lack clarity and are incomplete."

We thank you very much for your suggestion.

This study combined the optimization methods of surveying many injection parameters, applying mold temperature control, and using ANN tools for the weld line strength of PA6 samples.

We have modified the manuscript to clarify it and improve some sentences as follows:

*+ On page 5 lines 14-30 to read as:*

A novel approach to controlling the negative effect of the weld line is the utilization of high mold temperatures during molding, which significantly exceeds the common molding practice where temperatures are typically below 100°C. This is facilitated by a newly designed and manufactured mold structure that enables localized heating, termed the 'novel cavity', as introduced by Meister *et al.* [21]. However, the effects of injection parameters and especially mold temperature control on the weld line strength of PA6 are rarely discussed. The optimal parameters are also not fully considered.

This study delves into the impact of injection parameters and mold temperature control on the weld line strength of composite samples made of polyamide 6 (PA6). The potential improvements in weld line strength under elevated temperature conditions could be revealed. Additionally, this paper introduces a sophisticated method of optimization by integrating an artificial neural network (ANN) with a genetic algorithm (GA). This combination allows for a refined analysis of the molding parameters, taking the study beyond traditional experimental design and into the realm of predictive modeling and optimization. Furthermore, the research uses scanning electron microscopy (SEM) analysis to examine the fractures in the weld lines. The outcomes of this study could offer insights into enhancing the injection molding processes, where the optimization of weld line strength is paramount for the quality and durability of PA6 samples.

7. “The injection mold was designed and manufactured with the core and cavity plate as in Figure 2.” Verify the figure number.

We are sorry for this mistake, this is figure 3, not figure 2.

We have modified the manuscript as follows:

*+ On page 6 lines 4-5 to read as:*

The injection mold was designed and manufactured with the core and cavity plates, as presented in Figure 3

8. Why did the author choose not to employ a standard Design of Experiments (DOE) approach in parameter preparation?

We thank you very much for your suggestion.

Before beginning the optimization process, we tried to survey the reasonable parameters that could successfully inject the sample into a nice shape. The parameters in Table 1 were carefully selected from the previous survey to narrow the parameter range. It is simple, and the injection process could be controlled easily, as different samples could only require a single change in the parameters. We are going to apply the DOE method in further investigation based on the results of this study.

9. Indicate the results of UTS and %E in table 1 for better clarity.

We thank you very much for your suggestion.

We have added more information to Table 1.

We have modified the manuscript as follows:

*+ On page 9 lines 3-17 and page 10 line 1-9 to read as:*

**Table 1.** Injection molding parameters for PA6 samples.

| No. | Filling time (s) | Packing time  (s) | Melt temperature  (°C) | Mold temperature  (°C) | UTS (MPa) | Elongation (%) |
| --- | --- | --- | --- | --- | --- | --- |
| 1 | 3.0 | 0.4 | 244 | 145 | 36.47 | 225.26 |
| 2 | 3.2 |  |  |  | 39.17 | 193.34 |
| 3 | 3.4 |  |  |  | 49.93 | 394.35 |
| 4 | 3.6 |  |  |  | 43.3 | 365.95 |
| 5 | 3.8 |  |  |  | 45.53 | 341.97 |
| 6 | 3.4 | 0 | 244 | 145 | 45.03 | 397.52 |
| 7 |  | 0.2 |  |  | 37.53 | 248.52 |
| 8 |  | 0.4 |  |  | 49.93 | 394.35 |
| 9 |  | 0.6 |  |  | 51.23 | 410.94 |
| 10 |  | 0.8 |  |  | 55.37 | 543.96 |
| 11 | 3.4 | 0.4 | 240 | 145 | 38.7 | 254.68 |
| 12 |  |  | 242 |  | 53.5 | 532.47 |
| 13 |  |  | 244 |  | 49.93 | 394.35 |
| 14 |  |  | 246 |  | 55.67 | 549.33 |
| 15 |  |  | 248 |  | 40.8 | 307.57 |
| 16 | 3.4 | 0.4 | 244 | Without heating | 45.1 | 320.5 |
| 17 |  |  |  | 111 | 43.0 | 301.43 |
| 18 |  |  |  | 145 | 49.93 | 394.35 |
| 19 |  |  |  | 153 | 47.73 | 424.09 |
| 20 |  |  |  | 173 | 62.3 | 582.6 |

10. “The effects of filling time on the tensile test results are shown in Figs. 4, 5.” Correct it to only figure 5 as figure 4 only represent stress strain relationship.

We thank you very much for your suggestion.

We are sorry for this mistake; we have correct this sentence.

We have modified the manuscript as follows:

*+ On page 11 lines 5-6 to read as:*

The effects of filling time on the tensile test results are shown in Fig. 5.

11. “Packing time at 0.2s seems to not enough for an improvement in the UTS value. However, from 0.4s to 0.8s packing time, the UTS value of the PA6 sample experiences an increase compared to sample without packing step.” Why? Provide detailed explanation.

We thank you very much for your suggestion.

We have disscussed more about the 0.2s packing time. After checking the SEM results, we found that there are some voids, that could lead to the decrease of the mechanical properties in this sample.

We have modified the manuscript as follows:

*+ On page 22 lines 15-23 and page 23 lines 1-16 to read as:*

**Figure 14.** SEM picture of fracture surface of PA6 samples with weld line: (a1)‒(a2) sample 3, (b1)‒(b2) sample 7, (c1)‒(c2) sample 12, and (d1)‒(d2) sample 16.

Figure 14 illustrates the fracture surfaces of the PA6 samples with weld line at various magnifications. The SEM result shows the ductile fracture of the PA6 samples with the flat surface. The ductile fracture mechanism of the PA6 samples is the result of low stiffness, high elongation, and high energy absorption of the polymer [30, 31]. Moreover, there are some grooves on the surface, indicating the poor bonding structure of the weld line area. Interestingly, in sample 7, which has a packing time of 0.2 s, there is a void in the microstructure of the matrix, as shown in Figure 14(b2). The void causes a reduction in the polymer quality. Generally, applying extra packing time helps remove air bubbles. However, too short a packing time could cause an improper result due to the elastic deformation rather than the plastic deformation of the polymer sample after releasing the packing step. Therefore, the air bubble could be introduced again after releasing the packing step. This is the reason for the low UTS and elongation values of this sample, as shown in Figures 7–8. A packing time that is higher than 0.2s leads to a positive effect as the UTS and elongation values are improved.

[30] Vidakis, N., Petousis, M., Velidakis, E., Liebscher, M., Mechtcherine, V., & Tzounis, L. (2020). On the strain rate sensitivity of fused filament fabrication (Fff) processed pla, abs, petg, pa6, and pp thermoplastic polymers. *Polymers*, *12*(12), 2924.

[31] Taktak, R., Guermazi, N., Derbeli, J., & Haddar, N. (2015). Effect of hygrothermal aging on the mechanical properties and ductile fracture of polyamide 6: Experimental and numerical approaches. *Engineering fracture mechanics*, *148*, 122-133.

12. The parameter ranges, particularly for melt temperature, are limited to only 2°C. It appears that the defined ranges for each parameter may not be sufficient to draw conclusive results.

We thank you very much for your suggestion.

Before conducting the optimization process, we tried to survey the reasonable parameters that could successfully inject the sample with a good shape. Therefore, the parameter ranges are surveyed in a narrow range for better optimization results.

We have modified the manuscript to clarify as follows:

*+ On page 7 lines 4-6 to read as:*

Before conducting the optimization process, we tried to survey the reasonable parameters that could successfully inject the sample with a good shape. Therefore, the parameter ranges are surveyed in a narrow range for better optimization results.

13. Include details about the datasets used for training, validation, and testing. Additionally, clarify the strategy employed for selecting datasets for the same.

🡺 Thank you very much for your comment, here are the details about the datasets used for training, validation, and testing, as well as the strategy employed for selecting datasets.

**Datasets Used:**

- Total samples: 20 samples (including both PA6+30%GF and PA6+0%GF experimental samples)
- Data split:
  - Training: 70% (14 samples)
  - Validation: 15% (3 samples)
  - Testing: 15% (3 samples)

**Data Selection Strategy:**

- **Random Split:** The entire 20 samples were randomly divided into three sets: training, validation, and testing, in a 70:15:15 ratio.
- **Ensuring Diversity:** All three sets include samples from both PA6+30%GF and PA6+0%GF experiments to ensure data diversity and avoid bias during training.

**Rationale for Choosing this Strategy:**

- **Random Split:** Helps ensure objectivity and avoid biases in data selection.
- **70:15:15 Ratio:** This is a common ratio used in machine learning, providing sufficient data for all three sets: training, validation, and testing.
- **Ensuring Diversity:** Including samples from both experiments in each set helps the model learn complex relationships between input and output variables, thereby improving model accuracy.

In addition, above information was added into part 3.2 of the last version.

14. “First, we train the neural network with the input data, which is the UTS value of the samples.” Avoid the use of pronouns such as 'We' in the manuscript. Additionally, clarify whether UTS is considered input data.

We thank you very much for your suggestion.

We have removed “we” in the manuscript.

Moreover, the input and output data are also modified.

We have modified the introduction section as follows:

*+ On page 20 lines 15-23 to read as:*

First, the neural network is trained with the input data and output, which are injection parameters and the UTS value of the samples.

*+ On page 21 lines 1-2 to read as:*

GA (Genetic Algorithm) is used to optimize the results after training the neural network for the input and output data of PA6.

15. The modeling and optimization section lacks comprehensive understanding and information. For instance:

a. Crucial details regarding ANN modeling, such as architecture, ANN parameters, epochs, number of hidden layers, and hidden layer neurons, are omitted.

b. The specifics of GA and its parameters are not provided.

Thank you very much for your comment, the information of modeling and optimization were added in section 3.2 as:

🡺(a) details regarding ANN modeling

**Details regarding ANN modeling**

**Training Dataset:**

- The training dataset for the ANN model was extracted from the experimental data.
- The training/validation/testing dataset split was 75/15/15.

**ANN Architecture:**

- Number of hidden layers: 1
- Number of neurons in each hidden layer: 10

**Training Algorithm:**

- Levenberg-Marquardt algorithm

**Training Parameters:**

- Epochs: 8

🡺 (b) The specifics of GA and its parameters are

**1. Fitness Function:**

The fitness function is defined as follows:

2. Variable Bounds:

3. **Selection Technique:**

The selection technique used is Stochastic Uniform. Stochastic Uniform selection is a probabilistic selection method that randomly selects individuals from the population based on their fitness values. The probability of an individual being selected is proportional to its fitness value

4. **Crossover Technique:**

The crossover technique used is Intermediate crossover. Intermediate crossover is a type of single-point crossover that creates offspring by averaging the corresponding genes from the parent chromosomes

5. **Mutation Technique:**

The mutation technique used is Constraint-dependent mutation. Constraint-dependent mutation is a type of mutation that only modifies genes that violate the problem constraints. This helps to ensure that the offspring solutions remain feasible

6. **Stopping Criteria:**

The stopping criteria for the genetic algorithm are not explicitly stated in the provided text. It is recommended to check the original source or consult with the author to determine the stopping criteria used in the study

In addition, above information was added into part 3.2 of the last version

16. Table 3 does not adhere to the Pareto principle. The author should familiarize themselves with Pareto optimal solutions.

🡺 The name of table 3 was corrected

**Table 3.** The table of optimized results for PA6.

| PA6 | Filling time (s) | Packing time (s) | Melt temperature (^°^C) | Mold temperature (^°^C) |
| --- | --- | --- | --- | --- |
| ANN + GA | 3.8 | 0.8 | 244 | 35.2 |

17. In Table 4, clarify how the variation is calculated. Additionally, explain why the authors did not utilize a standard tool such as ANOVA to determine the influencing parameters.

We thank you very much for your suggestion.

We have removed Table 4 and applied a better tool to indicate the influence parameters.

We have modified the manuscript as follows:

*+ On page 21 lines 8-27 and page 22 lines 1-7 to read as:*

**Table 4.** Response Table for Means of UTS values.

| Level | Filling time | Packing time | Melt temperature | Mold temperature |
| --- | --- | --- | --- | --- |
| 1 | 36.47 | 45.03 | 38.70 | 45.10 |
| 2 | 39.17 | 37.53 | 53.50 | 43.00 |
| 3 | 48.15 | 46.25 | 46.28 | 45.56 |
| 4 | 43.30 | 51.23 | 55.67 | 47.73 |
| 5 | 45.53 | 55.37 | 40.80 | 62.30 |
| Delta | 11.68 | 17.84 | 16.97 | 19.30 |
| Rank | 4 | 2 | 3 | 1 |

**Table 5.** Response Table for Means of Elongation values.

| Level | Filling time | Packing time | Melt temperature | Mold temperature |
| --- | --- | --- | --- | --- |
| 1 | 225.3 | 397.5 | 254.7 | 320.5 |
| 2 | 193.3 | 248.5 | 532.5 | 301.4 |
| 3 | 405.2 | 368.7 | 365.4 | 366.6 |
| 4 | 365.9 | 410.9 | 549.3 | 424.1 |
| 5 | 342.0 | 544.0 | 307.6 | 582.6 |
| Delta | 211.9 | 295.4 | 294.7 | 281.2 |
| Rank | 4 | 1 | 2 | 3 |

Finally, the indication of which factors play the most important role is surveyed. The ANOVA analysis is applied via Minitab software. The response table for means of the PA6 samples with weld lines of the UTS and elongation values is shown in Tables 4 and 5. With the UTS value, the mold temperature factor has the highest rank, followed by the packing time and melt temperature factors, while the filling time has the lowest rank. Considering the elongation at break value, the packing time factor is the most important factor, followed by the melt temperature and mold temperature factors, while the filling time also has the lowest rank.

18. Figure 14 represents which sample? parameter setting?

We thank you very much for your suggestion.

We have added more information about Figure 14.

We have modified the manuscript as follows:

*+ On page 22 lines 15-23 to read as:*

**Figure 14.** SEM picture of fracture surface of PA6 samples with weld line: (a1)‒(a2) sample 3, (b1)‒(b2) sample 7, (c1)‒(c2) sample 12, and (d1)‒(d2) sample 16.

19. The author's classification of the fracture as ductile raises questions, as flat surfaces typically indicate brittle fractures. Further clarification is needed.

We thank you very much for your suggestion.

The surface is rough, indicating the ductility of the sample. We are sorry for this mistake.

We have modified the manuscript as follows:

*+ On page 22 lines 15-23 and page 23 lines 1-3 to read as:*

**Figure 14.** SEM picture of fracture surface of PA6 samples with weld line: (a1)‒(a2) sample 3, (b1)‒(b2) sample 7, (c1)‒(c2) sample 12, and (d1)‒(d2) sample 16.

Figure 14 illustrates the fracture surfaces of the PA6 samples with weld line at various magnifications. The SEM result shows the ductile fracture of the PA6 samples with the rough surface.

20. “The optimal molding parameters calculated from ANN and GA methods are a filling time of 3.8 s, a packing time is 0.8 s, a melt temperature of 244°C, and a mold temperature of 35.2 °C.” What are the corresponding values for UTS and %E? Has the author conducted any validation experiments?

🡺 In this paper, we focus on the method for mold temperature control, as well as the way to apply the ANN and GA to optimize the parameters, so, we just show out the optimization by the software. We hope that in the next research, the optimization will be applied in detail and the comparison between prediction and real experiment will be achieved.

*+ On page 23 to read as:*

This paper focuses on the method for mold temperature control, as well as the way to apply the ANN and GA to optimize the parameters. The study just shows the optimization by the software. In the next research, the optimization will be applied in detail and the comparison between prediction and real experiment will be achieved.
